# MicroRNA-370 inhibits the growth and metastasis of lung cancer by down-regulating epidermal growth factor receptor expression

**DOI:** 10.18632/oncotarget.21537

**Published:** 2017-10-04

**Authors:** Xin Liu, You-Guang Huang, Cong-Guo Jin, Yong-Chun Zhou, Xiao-Qun Chen, Jia Li, Yan Chen, Mei Li, Qian Yao, Ke Li, Min Lan, Jia-Gui Ye, Xi-Cai Wang

**Affiliations:** ^1^ Tumor Institute, The Third Affiliated Hospital of Kunming Medical University, Tumor Hospital of Yunnan Province, Kunming, China; ^2^ Pathological Department, The Third Affiliated Hospital of Kunming Medical University, Tumor Hospital of Yunnan Province, Kunming, China; ^3^ The Second Oncology Department, The Third Affiliated Hospital of Kunming Medical University, Tumor Hospital of Yunnan Province, Kunming, China

**Keywords:** lung cancer, mir-370, EGFR, proliferation, metastasis

## Abstract

Abnormal microRNA-370 (miR-370) expression has been frequently reported in several types of cancers, including lung cancer. However, the role and molecular mechanisms of miR-370 in regulating the growth and metastasis of lung cancer have not been clarified. Here, we show higher levels of epidermal growth factor receptor (EGFR), but lower levels of miR-370 expression in most human lung cancer cells and non-tumor cells. Induction of miR-370 over-expression significantly reduced the levels of EGFR expression and the EGFR 3′untranslated region (UTR)-regulated luciferase activity in XWLC-05 and H157 cells, suggesting that miR-370 may bind to the 3′UTR of EGFR mRNA. Compared with the control cells, induction of miR370 overexpression significantly inhibited the proliferation, clone formation capacity, migration and invasion of XWLC-05 and H157 cells while miR-370 inhibitor over-expression enhanced their tumor behaviors *in vitro*. Furthermore, miR-370 over-expression down-regulated the EGFR and hypoxia-inducible factor (HIF)-1α expression, and attenuated the extracellular single-regulated kinase (ERK)1/2 and AKT phosphorylation in XWLC-05 and H157 cells. In contrast, miR370 inhibitor over-expression increased the EGFR and HIF-1α expression as well as the ERK1/2 and AKT phosphorylation in XWLC-05 and H157 cells. Moreover, miR-370 over-expression significantly reduced the levels of EGFR and CD31 expression and inhibited the growth and lung metastasis of xenograft NSCLC tumors in mice. Our study indicates that miR-370 may bind to the 3′UTR of EGFR to inhibit EGFR expression and the growth, angiogenesis and metastasis of non-small cell lung cancer by down-regulating the ERK1/2 and AKT signaling.

## INTRODUCTION

Lung cancer is the leading cause of cancer-related deaths worldwide [1–3]. Patients with lung cancer have a 5-year survival rate of about 10~15% although diagnosis, surgical technique and new therapies for lung cancers have advanced during the past several years [4–6]. Notably, lung cancers are usually accompanied by the mutation of the epithelial growth factor receptor (EGFR) and its high expression [7–9]. High levels of EGFR expression can activate the downstream PI3K/AKT and MAPK/ERK to promote the proliferation and metastasis of lung cancers [10–12]. Hence, understanding the molecular pathogenesis of lung cancer, particularly for the high levels of EGFR expression-related signaling and development of new therapeutic strategies will be of great significance in management of patients with lung cancers.

MicroRNAs (miRNAs) are small non-coding RNA molecules of about 22 nucleotides [13, 14]. MiRNAs can bind to the complementary sites in the 3′-untranslated region (UTR) of its targeted genes to inhibit mRNA translation and promote mRNA degradation [15]. There are more than 1000 miRNAs [16], which will target about 60% of human genes [17] to regulate the biological process, such as proliferation, angiogenesis, migration and invasion and others of cancers [18]. Previous studies have shown that miR-138, miR-34 and miR-200c regulate the tumorigenesis and metastasis of lung cancers [19–21].

MiR-370 is located on human chromosome 14 (14q32), the DLK1-DIO3 genomic region [18]. A previous study has showed that miR-370 may have a causative role in the disorder of lipid metabolism [22]. Recent studies have revealed that miR-370 expression is downregulated in several types of cancer tissues, including bladder cancer [23], neuroblastoma [24], acute myeloid leukemia [25] and others. MiR-370 can function as a tumor suppressor by targeting the expression of FoxM1 [25, 26] and TRAF4 [27]. However, miR-370 expression is up-regulated in breast cancer and gastric carcinoma [28]. While one study indicates that miR-370 expression is associated with poor prognosis of patients with lung adenocarcinoma [29], another study reveals that induction of miR-370 over-expression inhibits the proliferation and promotes apoptosis of human lung cancer A549 and H358 cells [30]. Therefore, currently, the role of miR-370 in the tumorigenesis and metastasis of lung cancers remains controversial.

In this study, we examined the levels of miR370 and EGFR expression in several human lung cancer cell lines and non-tumor bronchial epithelial cells, and explored the effect of miR-370 on the proliferation, angiogenesis and migration of lung cancer cells *in vitro* and the growth and metastasis of lung cancers *in vivo*. We found that miR-370 acted as a tumor suppressor to reduce EGFR expression and inhibited the growth and metastasis of lung cancers by down-regulating the ERK1/2 and AKT signaling.

## RESULTS

### The EGFR and miR-370 expression in different lung cancer cells and non-tumor bronchial epithelial cells

**Figure 1:**
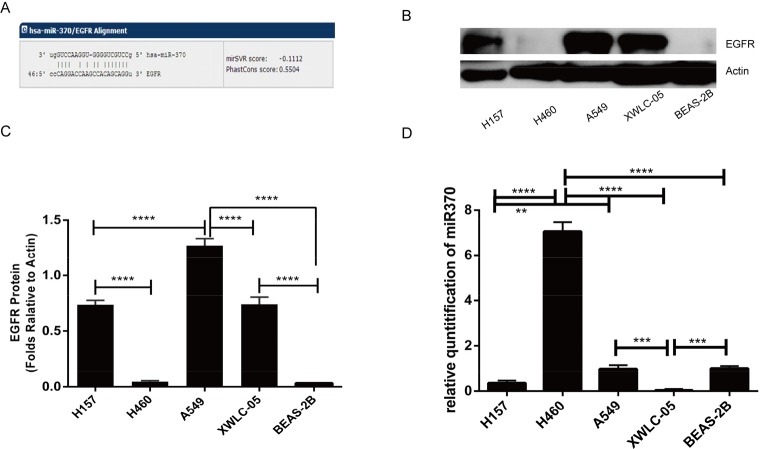
EGFR and miR370 expression in lung cancer cells **(A)** A potential binding site of miR-370 in the 3′UTR of the EGFR was predicted using online tools. **(B)** Western blot analysis of the relative levels of EGFR expression in the indicated cell lines. **(C)** Quantitative analysis of the levels of EGFR protein expression. **(D)** Real-time PCR detection of miR-370 transcripts. Data are representative images or expressed as the means ± SEM of each group from three separate experiments. ^**^P<0.01, ^***^P<0.001, ^****^P<0.0001.

The EGFR is encoded by the oncogene C-ERBB1 (HER-1) and is critical for the growth of malignant cells. However, the regulation on the EGFR expression is poorly understood. We found that the 3′UTR of the EGFR contained the potential binding site of miR-370 using several online database (http://www.microrna.org/microrna/home.do, http://www.targetscan.org/vert_71/) (Figure 1A). Next, we examined the relative levels of EGFR to control β-actin expression and miR-370 to U6 transcripts in human lung cancer A549, H460, H157, XWLC-05 cells, and non-tumor bronchial epithelial Beas-2b cells by Western blot and quantitative RT-PCR, respectively. High levels of EGFR expression were detected in A549, XWLC-05 and H157 cells, but much lower levels of EGFR were detected in large cell lung cancer H460 and non-tumor bronchial epithelial Beas-2b cells (Figure 1B and 1C). In contrast, high levels of miR-370 transcripts were detected in H460, Beas-2b, A549 and moderate levels of miR-370 in H157 cells while much lower levels of miR-370 in XWLC-05 cells (Figure 1D). Hence, the levels of miR-370 expression may be negatively associated with the levels of EGFR in these cells, except for A549 cells.

### Modulation of miR-370 expression affects the EGFR expression and the EGFR 3′UTR-regulated luciferase activity in lung cancer cells

**Figure 2:**
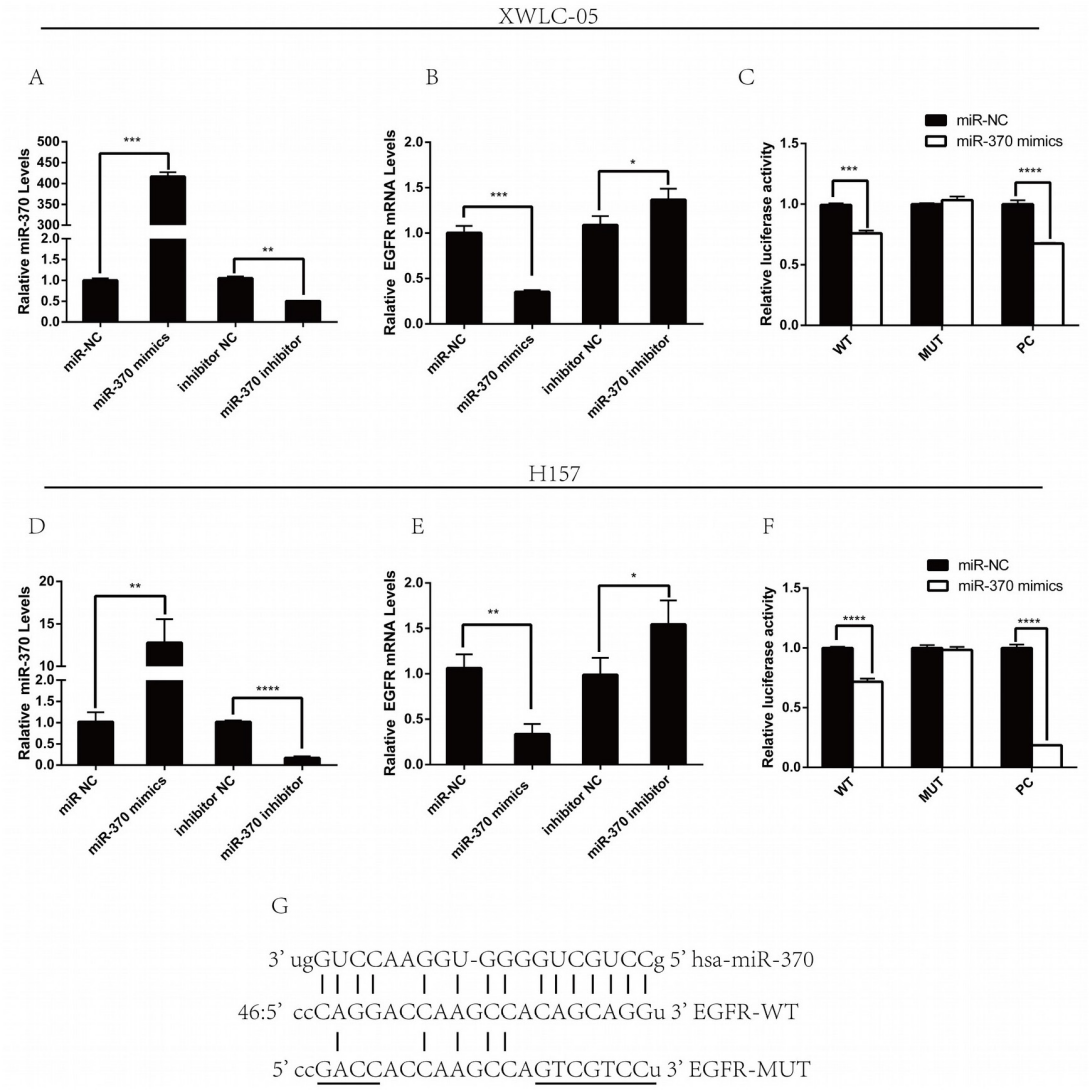
Modulation of miR-370 alters the EGFR expression in lung cancer cells XWLC-05 and H157 cells were transfected with miR-NC, miR-370 mimics, miR-370 inhibitor NC or miR-370 inhibitor for 24 h. The relative levels of miR-370 and EGFR mRNA transcripts in different groups of cells were determined by quantitative RT-PCR. In addition, XWLC-05 and H157 cells were co-transfected in triplicate with pmiRG-EGFR-UTRwt, pmiRG-EGFR-UTRmut or pmiRG-miR-370-inhibitor-PC, together with 20 nM miR-370 mimics or miR-NC for 48 h and the luciferase activities of individual cell samples were analyzed. Data are representative images or expressed as the means ± SEM of each group of cells from three separate experiments. **(A, D)** The levels of miR-370 transcripts. **(B, E)** The relative EGFR mRNA transcripts. **(C, F)** The luciferase activity. **(G)** Alignment of the sequences for miR-370 of binding motifs in the wild-type and mutated (underlined) 3′UTRs of EGFR for luciferase assays. ^*^P<0.05, ^**^P<0.01, ^***^P<0.001, ^****^P<0.0001.

To determine whether miR-370 regulates the EGFR expression, XWLC-05 and H157 cells were transfected with miR-370 mimics, miR-370 inhibitor, or their corresponding scrambled control. We found that transfection with miR-370 significantly increased levels of miR-370 expression, but decreased EGFR mRNA transcripts (Figure 2A and 2D) while transfection with miR-370 inhibitor significantly decreased the levels of miR-370 expression, but increased EGFR mRNA transcripts in both XWLC-05 and H157 cells, relative to their controls (Figure 2B and 2E). Next, we tested whether miR-370 affected the EGFR 3′UTR-regulated luciferase activity by dual-luciferase reporter assay. We synthesized the EGFR 3′UTR mutant at the potential binding sequence of miR-370 (Figure 2G) and generated pmiRG-EGFR-UTRwt, pmiRG-EGFR-UTRmu and pmiRG-miR-370-inhibitor-PC plasmids. Subsequently, we transfected XWLC-05 and H157 cells with pmiRG-EGFR-UTRwt, pmiRG-EGFR-UTRmu or pmiRG-miR-370-inhibitor-PC, together with miR-370 mimic or miR-NC for 48 h, respectively. In comparison with the controls transfected individual plasmids with miR-NC, transfection with miR-370 mimics, together with pmiRG-EGFR-UTRwt or pmiRG-miR-370-inhibitor-PC, but not pmiRG-EGFR-UTRmu significantly reduced the levels of luciferase activity in both XWLC-05 and H157 cells (Figure 2C and 2F). These suggest that miR-370 may bind to the 3′UTR of EGFR mRNA and the miR-370 inhibitor in both lung cancer cells.

### Modulation of miR-370 expression alters the neoplastic behaviors of lung cancer XWLC-05 and H157 cells *in vitro*

**Figure 3:**
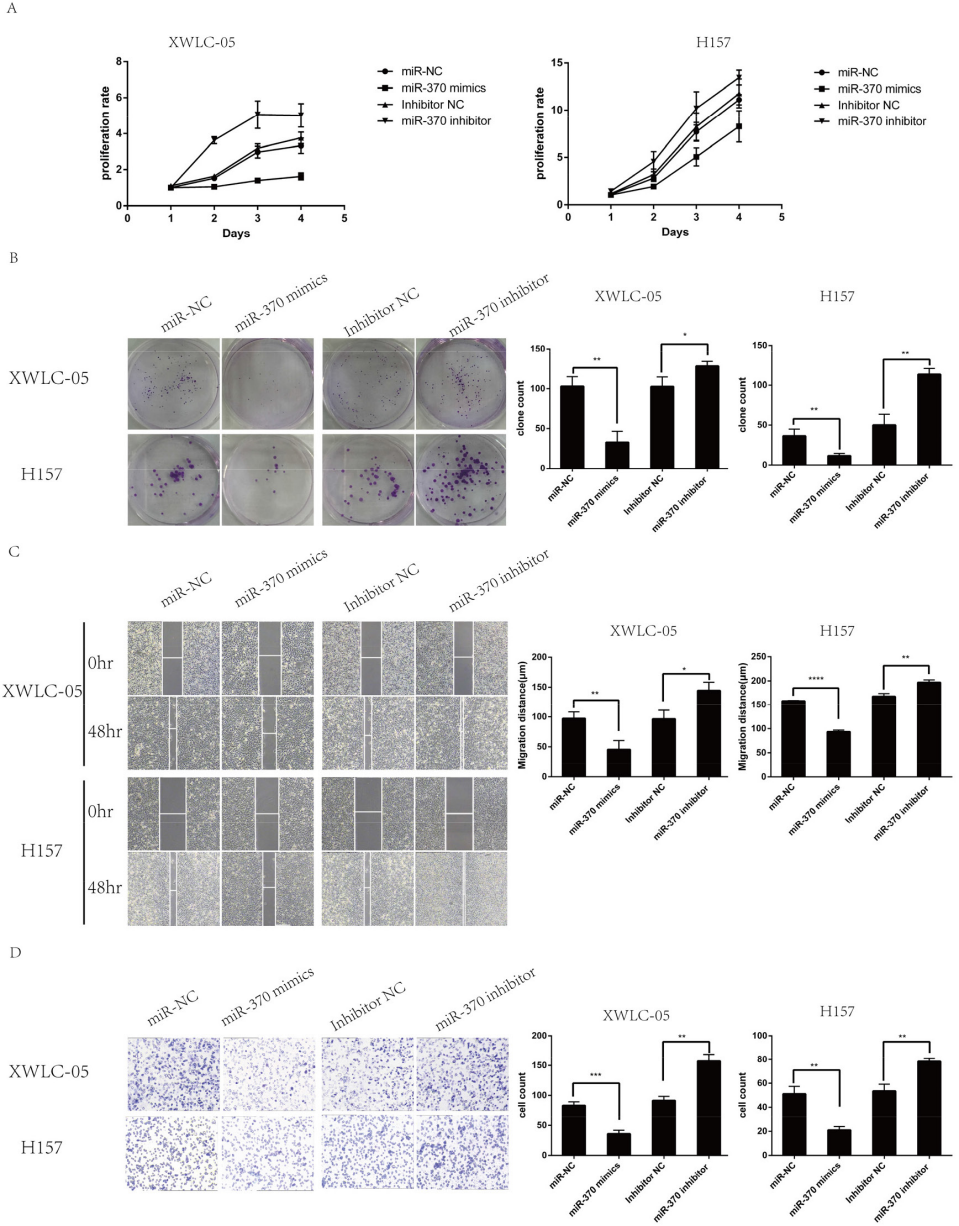
MiR-370 over-expression inhibits while inhibition of miR-370 expression enhances the proliferation, colony formation, wound healing and invasion of XWLC-05 and H157 cells XWLC-05 and H157 cells were transfected with miR-370 mimics, miR-370 inhibitor or their corresponding controls for 24 h. The proliferation, colony formation, wound healing and invasion of individual groups of cells were determined by MTT, colony formation, wound healing and transwell invasion assays, respectively. Data are representative images or expressed as the means ± SEM of each group of cells from three separate experiments. **(A)** The proliferation rates. **(B)** The colony formation. **(C)** Wound healing. **(D)** Invasion. ^*^P<0.05, ^**^P<0.01, ^***^P<0.001, ^****^P<0.0001.

To test the function of miR-370 in lung cancer cells, XWLC-05 and H157 cells were transfected with miR-370 mimics, miR-370 inhibitor, control miR-NC or control inhibitor and the dynamic proliferation of different groups of cells was determined by MTT assay. As shown in Figure 3A, induction of miR-370 over-expression dramatically reduced the proliferation rates while induction of miR-370 inhibitor expression enhanced the proliferation rats of XWLC-05 and H157 cells, relative to the control transfected with negative control. A similar pattern of colony formation, wound healing and invasion was detected among these groups of XWLC-05 and H157 cells (Figure 3B, 3C and 3D). Therefore, induction of miR-370 over-expression inhibited proliferation, clonogenicity, wound healing and invasion while inhibition of endogenous miR-370 by transfection with miR-370 inhibitor enhanced proliferation, clonogenicity, wound healing and invasion of XWLC-05 and H157 cells.

### Induction of miR-370 over-expression attenuates the EGFR expression and down-regulates the ERK and AKT signaling in XWLC-05 and H157 cells

**Figure 4:**
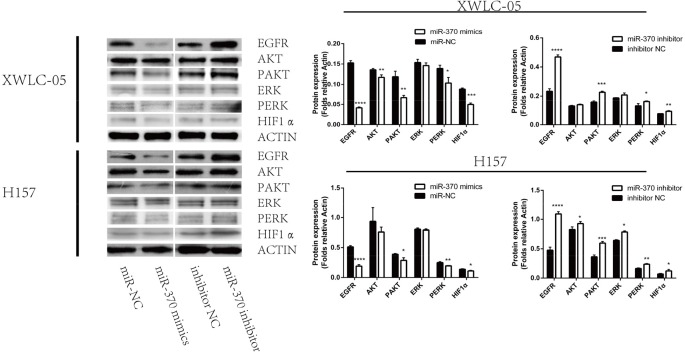
Induction of miR-370 over-expression reduces EGFR and HIF-1α expression and inhibits the ERK and AKT phosphorylation in XWLC-05 and H157 cells XWLC-05 and H157 cells were transfected with miR-370 mimics, miR-370 inhibitor or corresponding controls for 24 h. The relative levels of EGFR, HIF-1α, ERK, AKT expression, ERK and AKT phosphorylation were determined by Western blot assays. Data are representative images or expressed as the means ± SEM of each group of cells from three separate experiments. ^*^P<0.05, ^**^P<0.01, ^***^P<0.001, ^****^P<0.0001.

Engagement of EGFR by its ligand can activate the downstream ERK and PI3K/AKT signaling, which are crucial for the proliferation, migration and invasion of cancer cells. To explore the molecular mechanisms underlying the action of miR-370, XWLC-05 and H157 cell were transfected with miR-370 mimics, miR-370 inhibitor control miR-NC or control inhibitor. The relative levels of EGFR, ERK, AKT and HIF-1α expression, ERK and AKT phosphorylation were determined by Western blot assays. In comparison with the controls transfected with miR-NC, transfection with miR-370 mimics significantly reduced the relative levels of EGFR and HIF-1α expression, reduced AKT and ERK1/2 phosphorylation in XWLC-05 and H157 cells (Figure 4). In contrast, transfection with miR-370 inhibitor increased the relative levels of EGFR and HIF-1α expression, increased AKT and ERK1/2 phosphorylation in XWLC-05 and H157 cells. These independent lines of data indicated that miR-370 attenuated the EGFR-related ERK1/2 and AKT signaling in lung cancer cells *in vitro*.

### MiR-370 over-expression inhibits the growth and angiogenesis of xenograft NSCLC tumors *in vivo*

**Figure 5:**
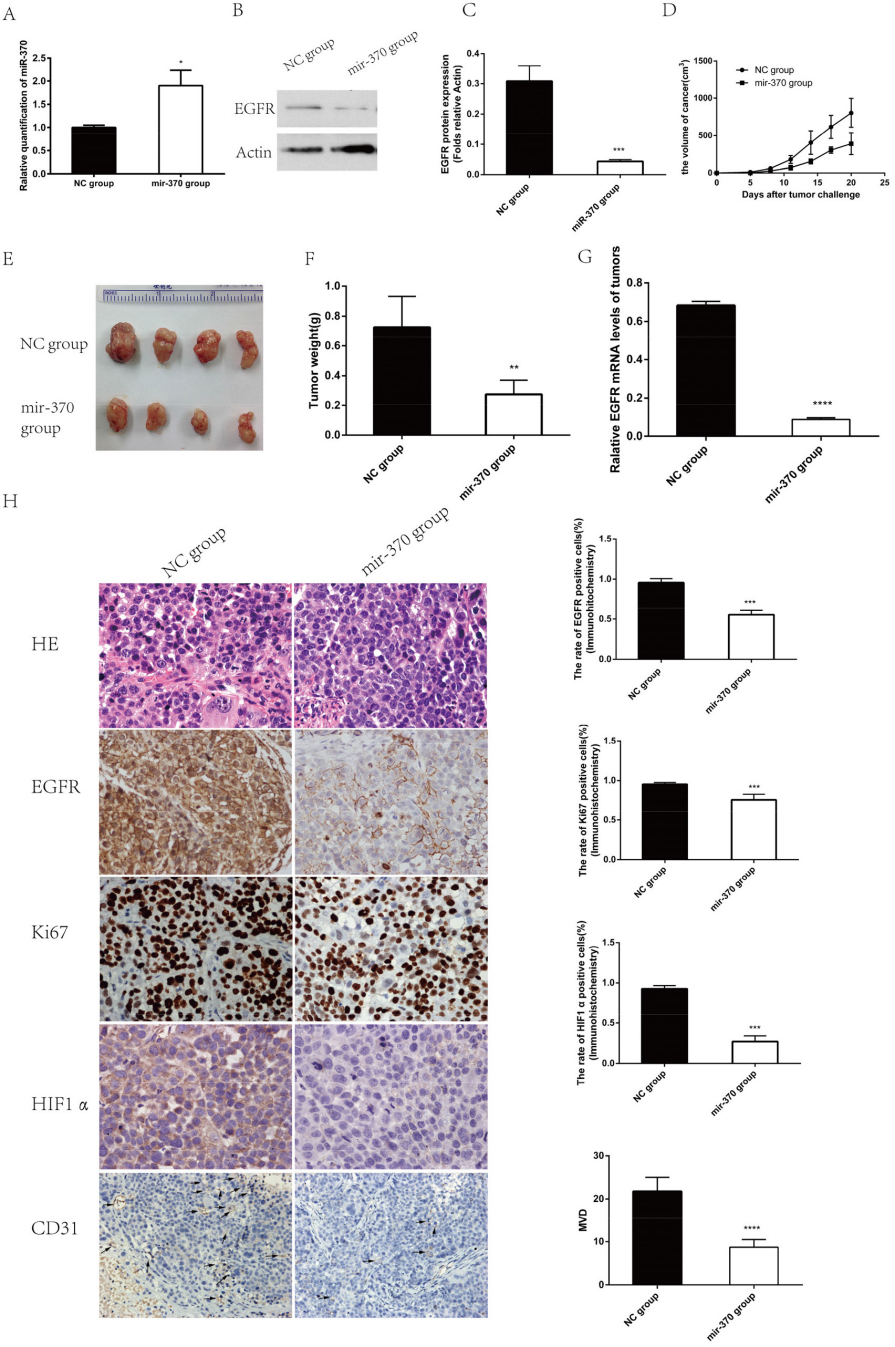
Induction of miR-370 over-expression inhibits the growth and angiogenesis of NSCLC xenograft tumors *in vivo* To establish a subcutaneous tumor model in nude mice, XWLC-05 cells were transfected with pre-miR-370 or miR-negative control (miR-NC). The cells were treated with 6 ng/mL blasticidin for 30 days to establish stable miR-370 over-expressing XWLC-05-miR-370 and control XWLC-05-miR-NC cells. The relative levels of miR-370 transcripts and EGFR expression in XWLC-05-miR-370 and control XWLC-05-miR-NC cells were determined by quantitative RT-PCR and Western blot assays, respectively. Individual female BALB/c nude mice were implanted subcutaneously with 2×10^6^ XWLC-05-miR-370 or XWLC-05-miR-NC cells (n=4, per group). The growth of formed solid tumors in individual mice were measured longitudinally up to 20 days post implantation. Their tumors were dissected, imaged and weighed. The relative levels of EGFR expression in individual rumor tissues were determined by quantitative RT-PCR. The tumor tissue sections were stained by H&E and the percentages of EGFR+ or Ki67+ tumor cells were determined by immunohistochemistry. In addition, the levels of HIF-1α and CD31 expression in the tumor sections were also examined by immunohistochemistry and the levels of MVD was determined. Data are representative images (magnification x 400 or 200) or expressed as the means ± SEM of each group. **(A)** The levels of miR-370 transcripts in the stable transfected cells. **(B)** Western blot analysis of EGFR protein expression in the stable transfected cells. **(C)** The levels of EGFR protein expression in the stable transfected cells. **(D)** The dynamic growth of implanted tumors in mice. **(E)** The dissected tumors. **(F)** The tumor weights. **(G)** The levels of EGFR mRNA transcripts in each group of tumors. **(H)** Histological and immunohistochemistry analysis. ^*^P<0.05, ^**^P<0.01, ^***^P<0.001, ^****^P<0.0001.

Next, we evaluated the effects of miR-370 on the growth of NSCLC xenografts *in vivo*. XWLC-05 cells were transfected with pre-miR-370 or miR-negative control (miR-NC) to establish stable XWLC-05-miR-370 over-expression and control XWLC-05-miR-NC cells. We found that XWLC-05-miR-370 cells displayed higher levels of miR-370, but lower EGFR expression than control XWLC-05-miR-NC cells (Figure 5A, 5B and 5C). Next, female BALB/c nude mice were implanted subcutaneously with 2×10^6^ XWLC-05-miR-370 or XWLC-05-miR-NC cells/mouse (4 mice per group). We found that the volumes of XWLC-05-miR-370 tumors were significantly smaller than XWLC-05-miR-NC tumors at day 11, 14, 17 and 20 post implantation (Figure 5D and 5E). At the end of the experiment, the XWLC-05-miR-370 tumor weights were significantly less than XWLC-05-miR-NC tumors (Figure 5F). Analysis of the tumor tissues indicated that the relative levels of EGFR mRNA transcripts in the XWLC-05-miR-370 tumors were significantly lower than XWLC-05-miR-NC tumors (Figure 5G). Immunohistochemistric analysis revealed that the percentages of EGFR+ or KI67+ cells and the levels of HIF-1α expression and microvessel density (MVD) in the XWLC-05-miR-370 tumor sections were significantly lower than that in the XWLC-05-miR-NC tumor sections (Figure 5H). These data demonstrated that induction of miR-370 expression significantly down-regulated the EGFR expression and inhibited the growth and angiogenesis of xenograft NSCLC tumors in mice.

### MiR-370 over-expression inhibits the lung metastasis of xenograft NSCLC tumors *in vivo*

**Figure 6:**
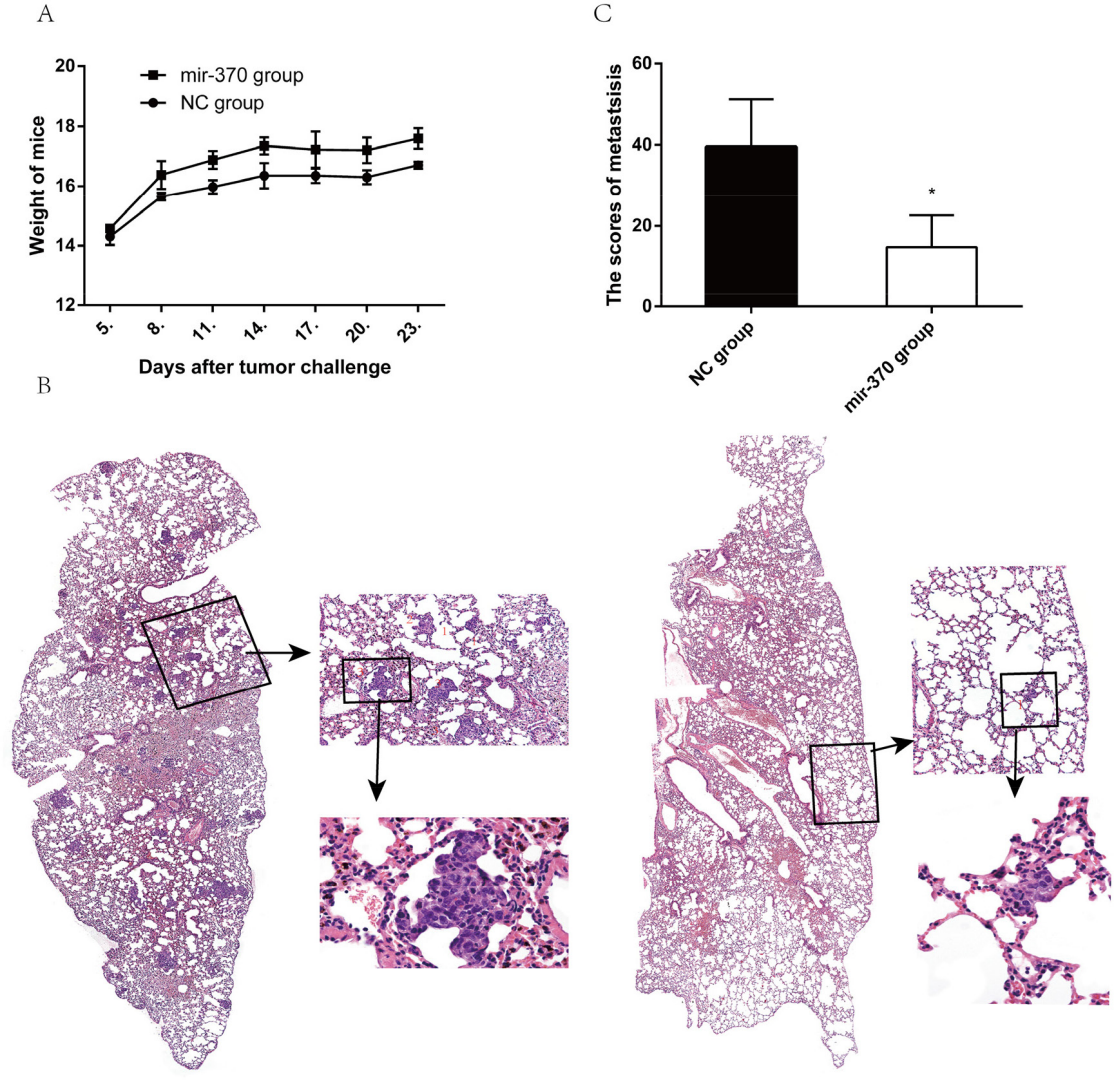
MiR-370 over-expression inhibits the lung metastasis of NSCLC xenografts *in vivo* Individual BALB/c nude mice were injected intravenously with 1×10^6^ XWLC-05-miR-370 or XWLC-05-miR-NC cells (n=4, per group). Four days later, their body weights were measured. At 24 days post injection, the mice were sacrificed and their lungs were dissected, followed by histological examination. Data are representative images (magnification x 100 or 400) or expressed as the means ± SEM of each group. **(A)** The body weights of mice. **(B)** Histological examination of the lungs. **(C)** The scores of tumor nodules in the lungs. ^*^P<0.05.

Finally, we examined the effect of miR-370 over-expression on the lung metastasis of xenograft NSCLC tumors *in vivo*. BALB/c nude mice were injected intravenously with XWLC-05-miR-370 or XWLC-05-miR-NC cells. Their body weights were measured every three days beginning at 4 days post injection. The body weights of the mice bearing XWLC-05-miR-370 tumors were significantly higher than that of those bearing XWLC-05-miR-NC tumors (Figure 6A). At 24 days post injection, all mice were sacrificed and their lung tissues were subjected to histological examination (Figure 6B). There were obviously more tumor nodules in the lungs of the mice bearing XWLC-05-miR-NC tumors than those bearing XWLC-05-miR-370 tumors. The micrometastases were scored according to the reference [32], and quantitative analysis revealed that the scores of lung metastatic tumors in the mice bearing XWLC-05-miR-370 tumors were significantly less than those bearing XWLC-05-miR-NC tumors (Figure 6C). Therefore, induction of miR-370 over-expression inhibited the lung metastasis of xenograft NSCLC tumors in mice.

## DISCUSSION

The EGFR-related signaling is crucial for the development of tumors and is a therapeutic target for treatment of cancers [33–35]. In this study, we found that the 3′UTR of the EGFR contained a potential binding site of miR-370 and the levels of EGFR were negatively associated with the levels of miR-370 expression in several human lung cancer cell lines and non-tumor bronchial epithelial cells. These consistent with previous findings and support the notion that miR-370 is a tumor suppressor of cancer [36–38]. Furthermore, we found that induction of miR-370 over-expression attenuated the EGFR expression and inhibited the proliferation, clonogenicity, and invasion of lung cancer cells while inhibition of endogenous miR-370 by transfection with miR-370 inhibitor significantly enhanced the EGFR expression and the proliferation, clonogenicity, and invasion of lung cancer cells. More importantly, transfection with miR-370 significantly reduced the EGFR 3′UTR-wt-regulated luciferase activity, but did not affect the EGFR 3′UTR-mut-regulated luciferase activity in both XWLC-05 and H157 cells. These independent experimental results suggest that miR-370 may bind to the 3′UTR of the EGFR mRNA to inhibit EGFR expression. We are interested in further determining whether miR-370 directly binds to the 3′UTR of the EGFR to understand the precise role and mechanisms in regulating the EGFR expression in lung cancer.

Engagement of the EGFR by its ligand can activate the downstream MAPK and AKT signaling to promote the proliferation, angiogenesis and metastasis of lung cancers [39, 40]. In this study, we found that miR-370 over-expression inhibited the proliferation, clonogenicity, wound healing and invasion of XWLC-05 and H157 cells *in vitro*. Furthermore, miR-370 over-expression not only attenuated the EGFR and HIF-1α expression, but also significantly reduced the relative levels of ERK1/2 and AKT activation in XWLC-05 and H157 cells. In contrast, inhibition of endogenous miR-370 by transfection with miR-370 inhibitor significantly enhanced the proliferation, clonogenicity, wound healing and invasion in XWLC-05 and H157 cells, accompanied by increased levels of EGFR and HIF-1α expression as well as the ERK1/2 and AKT activation. More importantly, miR-370 over-expression inhibited the growth, angiogenesis and metastasis of implanted lung tumors in mice, accompanied by attenuating EGFR and Ki67 expression as well as MVD in the tumors. The reduced EGFR and Ki67 expression in the implanted tumors further supported that miR-370 over-expression inhibited proliferation of lung cancer cells *in vitro*. Given that the ERK and AKT signaling are important for the growth, angiogenesis and metastasis of cancers the inhibition of lung cancer growth, metastasis and angiogenesis by miR-370 over-expression may be also mediated by miR-370 attenuating the EGFR expression and down-stream ERK and AKT signaling *in vivo*. Our findings extended a previous report that miR-370 inhibits the progression of NSCLC by targeting the TRAF4 [30], but disagreed with a previous report that miR-370 expression is associated with poor prognosis of lung cancers [29]. The discrepancy may stem from the difference between our cell line study and their clinical study. Previous studies have shown that miR-370 can promote liver cancer cell apoptosis by targeting the AKT signaling and inhibits glioma cell proliferation and bladder cancer metastasis by targeting the β-catenin [38, 41, 42]. These, together with observation of down-regulated miR-370 expression in most lung cancer cells tested, suggest that miR-370 may inhibit the development and progression of lung cancers. We are interested in further investigating its expression and function in clinical lung cancer samples.

In summary, our data suggest a negative association between the levels of EGFR and miR-370 expression in human lung cancer cell lines and non-tumor bronchial epithelial cells. Induction of miR-370 over-expression attenuated the EGFR expression while inhibition of endogenous miR-370 expression by transfection with miR-370 inhibitor increased the EGFR expression in lung cancer cells. MiR-370 over-expression significantly reduced the EGFR 3′UTR-regulated luciferase activity, suggesting that miR-370 may bind to the 3′UTR of the EGFR to inhibit the EGFR expression in lung cancer cells. More importantly, miR-370 over-expression inhibited the proliferation, clonogenicity, wound healing and invasion of lung cancer cells *in vitro*, which were associated with reduced levels of EGFR and HIF-1α expression and the ERK1/2 and AKT activation. MiR-370 over-expression attenuated the growth, angiogenesis and metastasis of implanted lung tumors *in vivo*. Therefore, miR-370 may be a tumor suppressor and our findings may aid in design of new therapeutic strategies for intervention of lung cancers.

## MATERIALS AND METHODS

### Cell culture

Human lung cancer A549, H460, H157, XWLC-05 cells and a human immortalized non-tumor bronchial epithelial Beas-2b cells were obtained from Yunnan Cancer Institute, China. A549, H460, H157 and XWLC-05 cells were cultured in RPIM1640 while Beas-2b cells were cultured in Dulbecco's modified Eagle's medium (DMEM) (Gibco, New York, USA) containing 10% fetal bovine serum (FBS, Biosera, Nuaile, France), 100 Units/ml penicillin and 100 μg/ml streptomycin (complete medium) at 37°C in a humidified atmosphere of 5% CO_2_.

### Transfection with miR-370 mimics, inhibitors

XWLC-05 and H157 cells were cultured in medium without antibiotics overnight and transfected with 20 nM miR-370 mimics, 40 nM miR-370-inhibitor, or their corresponding scrambled controls for 24 hours using the Lipofectamine TM 2000 transfection reagent (Invitrogen). The transfection efficacy was determined by quantitative RT-PCR. The cells were used for following experiments.

### Reverse transcription polymerase chain reaction

Total RNA were extracted from individual types of cells using Trizol reagent (Life Technologies, Carlsbad, USA) and reversely transcribed into cDNA using the Hairpin-it^TM^ miRNAs qPCR quantitation kit (Genepharma, Shanghai, China) or the first strand cDNA synthesis kit (Takara, Dalian, China), according to the manufacturers’ instructions. Subsequently, the relative levels of miR-370, EGFR to the control U6 and rps13 transcripts in individual samples were determined in triplicate by real-time PCR using the specific primers. The sequences of primers were forward 5′- GCCTCCAGAGGATGTTCAATAA-3′ and reverse 5′-TGAGGGCAATGAGGACATAAC-3′ for EGFR (132 bp); forward 5′-GTTGCTGTTCGAAAGCATCTTG-3′, and reverse 5′-AATATCGAGCCAAACGGTGAA-3′ for rsp13 (100 bp); forward 5′-TAGCCTGCTGG GGTGGAA-3′ and reverse 5′- TATGGTTTTGACG ACTGTGTGAT-3′ for miR-370 (73 bp); forward 5′-ATTGGAACGATACAGAGAAGATT-3′ and reverse 5′-GGAACGCTTCACGAATTTG-3′ for U6 (81 bp). The data were normalized to the rsp13 or U6 and analyzed using 2^-ΔΔCt^ [31].

### Western blot

The different groups of cells were washed twice with cold PBS, and lyzed in RIPA lysis buffer (Beyotime Biotechnology Jiangsu, China) containing phosphatase and protease inhibitor cocktail (Roche, USA) for 30 min, followed by centrifugation. After the protein concentrations were quantified using the BCA kit (Beyotime Biotechnology), the cell lysates (30 μg/lane) were separated by sodium dodecyl sulfate polyacrylamide gel electrophoresis (SDS-PAGE) on 12% gels, and transferred onto polyvinylidene fluoride (PVDF) membranes (Millipore, USA). The membranes were blocked with 5% fat-free dry milk in TBST and incubated overnight at 4°C with rabbit antibodies, including anti-HIF-1α, anti-phos-Erk, anti-Erk1/2, anti-EGFR (1:1000; Affinity Biosciences, USA), anti-phos-Akt, anti-Akt (1:1000; Cell Signaling Technology, MA, USA) or anti-β-actin (1:4000; Sigma, USA). The bound antibodies were detected with horseradish peroxidase-conjugated goat anti-rabbit IgG (1:4000; Santa Cruz Biotechnology, CA, USA) and visualized using the enhanced *chemiluminescence* (ECL-plus) reagents (Merck Millipore, USA). The relative levels of targeted protein to the control β-actin expression were determined by densitometric analysis using the Image J software (USA).

### Dual-luciferase reporter assay

For luciferase reporter assay, oligonucleotides for the EGFR 3′-UTR wild type (WT), EGFR 3′-UTR mutant and Hsa-miR-370-3P inhibitor (as a positive control) were synthesized by Genepharma (Shanghai, China). After being annealed, the DNA fragments were cloned at the SacI/XhoI sites of the pmiRGLO firefly luciferase-expressing vector (Promega, WI, USA) to generate plasmids of pmiRG-EGFR-UTR-wt, pmiRG-EGFR-UTR-mut and pmiRG-miR-370-inhibitor-PC, respectively, followed by sequencing. XWLC-05 and H157 cells (1×10^5^/well) were cultured in 24-well plates overnight and co-transfected in triplicate with 250 ng pmiRG-EGFR-UTRwt, pmiRG-EGFR-UTRmu or pmiRG-miR-370-inhibitor-PC, together with 20 nM miR-370 mimic or miR-NC, by Lipofectamine 2000 according to the manufacturer's instructions. Forty-eight h after transfection, the luciferase activities of individual cell samples were analyzed using the Dual Luciferase Reporter Assay System (Promega, USA), following the manufacturer's protocol in Tecan M1000.

### Cell proliferation and colony formation assays

XWLC-05 and H157 cells were transfected with miR-370 mimics, miR-370 inhibitor, or corresponding scrambled controls for 24 h. The different groups of cells (10^4^ cells/well) were cultured in 96-well plates for 4 days and the cell viability of each group of cells was determined in triplicate using 3-(4, 5-dimethyl-thiazol-2-yl) 2, 5-diphenyltetrazolium bromide (MTT) reagent (Sigma). During the last 4-h incubation of each day, the cells in individual wells were exposed to 20 μl MTT reagent (5 mg/ml) and the resulting formazan in individual wells was dissolved in 150 μl of dimethylsulfoxide (DMSO), followed by measuring the absorbance at 490 nm using a Multiskan spectrum (Thermo).

The impact of miR-370 on the clonogenicity of tumor cells was determined by colony formation assay. Briefly, the different groups of cells (500 cells/well) were cultured in triplicate in a 6-well-plate for 7-10 days. The formed cell clones in each well were stained with 1 ml of 0. 5% crystal violet for 30 minutes and the numbers of cell clones were counted in a blinded manner.

### Wound healing and transwell invasion assays

The impact of miR-370 on the wound healing of tumor cells was determined by wound healing assay. Briefly, the different groups of cells (1×10^6^ cells/well) were cultured in 6-well plates for 24 h to obtain an 80% confluency. The monolayer of cells was scratched in triplicate using a P200 pipette tip, and cultured for 48 h. The distances between the edges of the scratch were measured and the net migration distance (initial distance - the final distance) of each well was quantitatively evaluated.

The impact of miR-370 on the invasion of tumor cells was determined by transwell matrigel invasion assay. Briefly, the different groups of cells (2×10^5^ cells/well) were serum-starved for 24 h and cultured in the upper chamber of Transwell plates (8-mm pore size; Corning) that had been coated with Matrigel (BD Biosciences). The bottom chambers were added with 600 μl RPMI 1640 medium containing 20% FBS and cultured for 24 h. The cells on the surface of the upper chamber membrane were removed by cotton swabs and the invaded cells on the bottom surface of the upper chamber membranes were stained with 0.1% crystal violet. The numbers of invaded cells were counted under a phase contrast microscope in a blinded manner.

### *In vivo* tumor xenograft assay

To establish stable transfected cells, pre-miR-370 or miR-negative control (miR-NC) were cloned into the plasmid pPG/miR/EGFP (Genepharma). To induce miR-370 over-expression, XWLC-05 cells were transfected with pPG/miR-370/EGFP or control pPG/miR-NC/EGFP using Lipofectamin^TM^ 2000 reagent for 24 h. The cells were treated with 6 ng/mL of blasticidin S (Sigma) for 30 days to establish stable XWLC-05-miR-370 over-expression and control XWLC-05-miR-NC cells. The levels of miR-370 expression were determined by quantitative RT-PCR and of EGFR expression were determined by Western-blot.

Female BALB/c nude mice at 5 weeks of age were purchased from Vital River (Charles River China, Beijing, China) and housed in a specific pathogen-free facility with free access to autoclaved food and water at the Traditional Chinese Medicine Hospital in Yunnan province. All animal care and handling procedures were performed in accordance with the National Institutes of Health's Guide for the Care and Use of Laboratory Animals and the experimental protocol was approved by the Institutional Ethic Committee of the Third Hospital Affiliated Kunming Medical University.

Individual mice were implanted subcutaneously with 2×10^6^ XWLC-05-miR-370 or XWLC-05-miR-NC cells (4 mice per group). The growth of formed solid tumors and body weights of individual mice were measured every three days up to 20 days post implantation. The volumes of tumors were calculated as follows: volume = (a×b^2^)/2, where a means the larger tumor diameter and b for the perpendicular diameter. At the end of the experiment, all of mice were sacrificed and their tumors were dissected, imaged and weighed. The relative levels of EGFR mRNA transcripts in the tumors were determined by quantitative RT-PCR and the tumor sections were examined by histology and immunohistochemistry.

The impact of miR-370 on the metastasis of implanted tumors was determined. Briefly, individual mice were injected intravenously with 1×10^6^ XWLC-05-miR-370 or XWLC-05-miR-NC cells (4 mice per group). Their body weights were measured every 3 days beginning on day 4 up to 24 days post injection. The mice were sacrificed and their lungs were dissected. The dissected lung tissues from individual mice were fixed in 10% buffered formalin and paraffin-embedded. The lung tissue sections (4 μm) were stained with hematoxylin and eosin, and photoimaged under a light microscope (Leica DM4000B, Solms, Germany). The images were evaluated by a pathologist (at Department of Pathology, the Third Hospital Affiliated Kunming Medical University) in a blinded manner. The pulmonary micrometastases were scored, according to the size (S) of the majority of lesions as: 1, small lesions containing approximately 25-100 tumor cells; 2, medium-sized lesions containing approximately 100-500 tumor cells; 3, large lesions containing more than 500 tumor cells [32]. The lung involvement was also scored for the surface area (A) interested by lesions: 1, < 5% of the lung surface involved; 2, between 5% and 50% of the lung surface involved; 3, >50% of the lung surface involved. A metastatic score was obtained by multiplying these two partial scores as size × involved area (S × A).

### Immunohistochemistry

The levels of EGFR, HIF-1α, Ki67 and CD31 expression in the tumor tissues were determined by immunohistochemistry. Briefly, the tumor sections (4 μm) were rehydrated, treated with 3% H_2_O_2_ in methanol and subjected to antigen retrieval using ready-to-use target retrieval solution (Maixin Biotech, Fuzhou, China). The sections were treated with 3% BSA and after being washed, the sections were incubated with anti-EGFR, anti-HIF-1α, anti-Ki67 or anti-CD31 (Affinity Biosciences, Cincinnati, USA) overnight at 4°C. Negative control sections were incubated with isotype-matched Ig. The bound antibodies were detected with HRP-conjugated second antibodies and visualized using the Elivision DAB kit (Maixin Biotech, Fuzhou, China), followed by photoimaging (magnification x 200 or 400). The percentages of EGFR+ or Ki67+ tumor cells were counted and the intensity of positive signals in three fields of each section (n = 4 per group) was evaluated using the Image J software.

### Statistical analysis

All statistical analyses were performed using the SPSS 17.0 (SPSS Software, USA). Data are representative images or expressed as mean ± standard error of the mean (S.E.M). The difference between the groups was determined by Student's T test. A *P*-value of < 0.05 was considered statistically significant.

## Abbreviations

miR-370: microRNA-370
EGFR: epidermal growth factor receptor
MTT: 3-(4, 5-dimethyl-thiazol-2-yl) 2, 5-diphenyltetrazolium bromide
